# Tunable Low‐Pressure Water Adsorption in Stable Multivariate Metal‐Organic Frameworks for Boosting Water‐Based Ultralow‐Temperature‐Driven Refrigeration

**DOI:** 10.1002/advs.202308623

**Published:** 2024-01-15

**Authors:** Chen‐Han Guo, Feng‐Fan Lu, Enyu Wu, Jia‐Xin Wang, Defa Gu, Bin Li, Guodong Qian

**Affiliations:** ^1^ State Key Laboratory of Silicon and Advanced Semiconductor Materials School of Materials Science and Engineering Zhejiang University Hangzhou 310027 China

**Keywords:** adsorption chillers, metal‐organic frameworks, multivariate strategy, refrigeration, water adsorption

## Abstract

The green water‐based adsorption refrigeration is considered as a promising strategy to realize near‐zero‐carbon cooling applications. Although many metal‐organic frameworks (MOFs) have been developed as water adsorbents, their cooling performance are commonly limited by the insufficient water uptakes below *P*/*P*_0_ = 0.2. Herein, the development of multivariate MOFs (MTV‐MOFs) is reported to highly modulate and boost the low‐pressure water uptake for improving coefficient of performance (COP) for refrigeration. Through ligand exchange in the pristine MIL‐125‐NH_2_, a series of MTV‐MOFs with bare nitrogen sites are designed and synthesized. The resulting MIL‐125‐NH_2_/MD‐5% exhibits the significantly improved water uptake of 0.39 g g^−1^ at 298 K and *P*/*P*_0_ = 0.2, which is three times higher than MIL‐125‐NH_2_ (0.12 g g^−1^) and comparable to some benchmark materials including KMF‐1 (0.4 g g^−1^) and MIP‐200 (0.36 g g^−1^). Combined with its low‐temperature regeneration, fast sorption kinetics and high stability, MIL‐125‐NH_2_/MD‐5% achieves one of the highest COP values (0.8) and working capacities (0.24 g g^−1^) for refrig‐2 under an ultralow‐driven temperature of 65 °C, which are higher than some best‐performing MOFs such as MIP‐200 (0.74 and 0.11 g g^−1^) and KMF‐2 (0.62 and 0.16 g g^−1^), making it among the best adsorbents for efficient ultralow‐temperature‐driven refrigeration.

## Graphical abstract

A novel and stable multivariate MOF (MIL‐125‐NH_2_/MD‐5%) is reported, constructed by the multivariate strategy, exhibits the significantly improved low‐pressure water uptake of 0.39 g g^−1^ at 298 K and *P*/*P*_0_ = 0.2, affording by far the top‐tier coefficient of performance (0.8) and working capacity (0.24 g g^−1^) for refrig‐2 under an ultralow driving temperature of 65 °C.

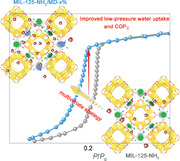

## 1 Introduction

Global warming has largly increased the energy and cost requirements for cooling systems in both industrial production and daily life.^[^ ^1^, ^2^ ^]^ Current cooling systems mainly depend on non‐sustainable energy resources derived from fossil fuels, wherein the huge electricity consumption along with greenhouse gas emission pose a significant global problem.^[^ ^3^, ^4^ ^]^ The development of more sustainable and energy‐efficient cooling systems by using clean and renewable energy resources has become of utmost importance. Water‐based adsorption chillers (ACs) have been considered as a potential low‐carbon refrigeration system due to the potential use of renewable solar energy or low‐grade heat.^[^ ^5^, ^6^, ^7^, ^8^ ^]^ Since this technology consists of a working cycle and a regeneration cycle based on a full cycle of water adsorption/desorption (Figure S1, Supporting Information), the cooling efficient is thus highly dependent on the adsorption and desorption amount of water vapor.^[^ ^9^, ^10^, ^11^, ^12^ ^]^

Depending on the temperature required, cooling can be subdivided into three different applications (Table S1, Supporting Information). For cooling in refrig‐1 region (283 K), the adsorbents should have a maximum water uptake (*W*_max_) at adsorption potential *A* = 3.12 kJ mol^−1^, and at 3.99 kJ mol^−1^ for cooling in refrig‐2 region (278 K).^[^ ^9^ ^]^ The case of ice making (refrig‐3) needs alternatives for water, such as methanol or ammonia. Corresponding to water adsorption curves, refrig‐1 and refrig‐2 require adsorbents to have large adsorption amounts at *P*/*P*_0_ = 0.30 and 0.20, respectively. Therefore, to achieve high refrig‐2 cooling performance, the adsorbents should meet the following criteria: i) S‐shaped water adsorption isotherms and with high working uptake at *P*/*P*_0_ < 0.2; ii) high performance coefficient (COP); iii) fast water adsorption and desorption kinetics; iv) low regeneration temperature; v) excellent water and cycling stability.

Water adsorbents play the vital factor to determine the high efficiency of this cooling system. Conventional water adsorbents (e.g., silica gels and zeolites) have been extensively reported for sorption‐based heating and cooling systems. However, there commonly exist clear drawbacks for these materials, including high regeneration temperatures for zeolites (>150 °C) and low working capacities for silica gels at low relative humidity, resulting in poor cooling performance and energy efficiency for water‐based adsorption systems.^[^ ^13^, ^14^, ^15^ ^]^ Alternatively, microporous metal‐organic frameworks (MOFs) have demonstrated to be an emerging type of promising water adsorbents due to the powerful designability and tunability on pore size and functionality.^[^ ^16^, ^17^, ^18^, ^19^, ^20^, ^21^ ^]^ A variety of MOFs have been recently developed as outstanding adsorbents for water sorption applications.^[^ ^22^, ^23^, ^24^, ^25^, ^26^, ^27^, ^28^, ^29^ ^]^ However, most of the reported MOFs still suffer from insufficient water uptakes below *P*/*P*_0_ = 0.2 due to the intrinsic contradiction between pore volume and low‐pressure water uptake. For instance, those large‐pore MOFs (e.g., Cr‐soc‐MOF‐1 and MIL‐101) show ultrahigh water uptake that is commonly ocurred at high relative humidity, while their weak water affinities limit their uptake amount at low relative pressure.^[^ ^30^, ^31^ ^]^ Although some small‐pore MOFs can capture water at *P*/*P*_0_ = 0.2 due to the strong water affinity, their low pore volumes largely limit the water uptakes, as demonstrated by MIL‐160 and CAU‐10.^[^ ^32^, ^33^, ^34^ ^]^ To enhance water adsorption at low‐pressure regions, the most popular strategy reported is to incorporate hydrophilic groups (e.g., ─OH and ─NH_2_) into porous MOFs to increase pore hydrophilicity and thus water binding affinity.^[^ ^35^, ^36^ ^]^ However, such increased water binding affinity was commonly targeted at the expense of a notable drop in uptake capacity in most cases, since the immobilized bulky functional groups result in an obvious decrease in pore volume, as exemplified in the ─OH and ─NH_2_ functionalized CAU‐10 and UiO‐66.^[^ ^36^, ^37^, ^38^, ^39^ ^]^ On the other hand, the hydrophilic groups are often difficult to be fully incorporated into MOFs to design the desired functionalized MOFs with the maintenance of high crystalline and high purity, making this strategy unsuccessful in most cases to improve low‐pressure water uptake. These limitations make the design of new adsorbents with high low‐pressure water uptakes become very challenging, and only limited MOFs have shown high water uptakes exceeding 0.35 g g^−1^ at *P*/*P*_0_ = 0.2 and 298 K for AC applications.^[^ ^40^, ^41^, ^42^ ^]^

**Figure 1:**
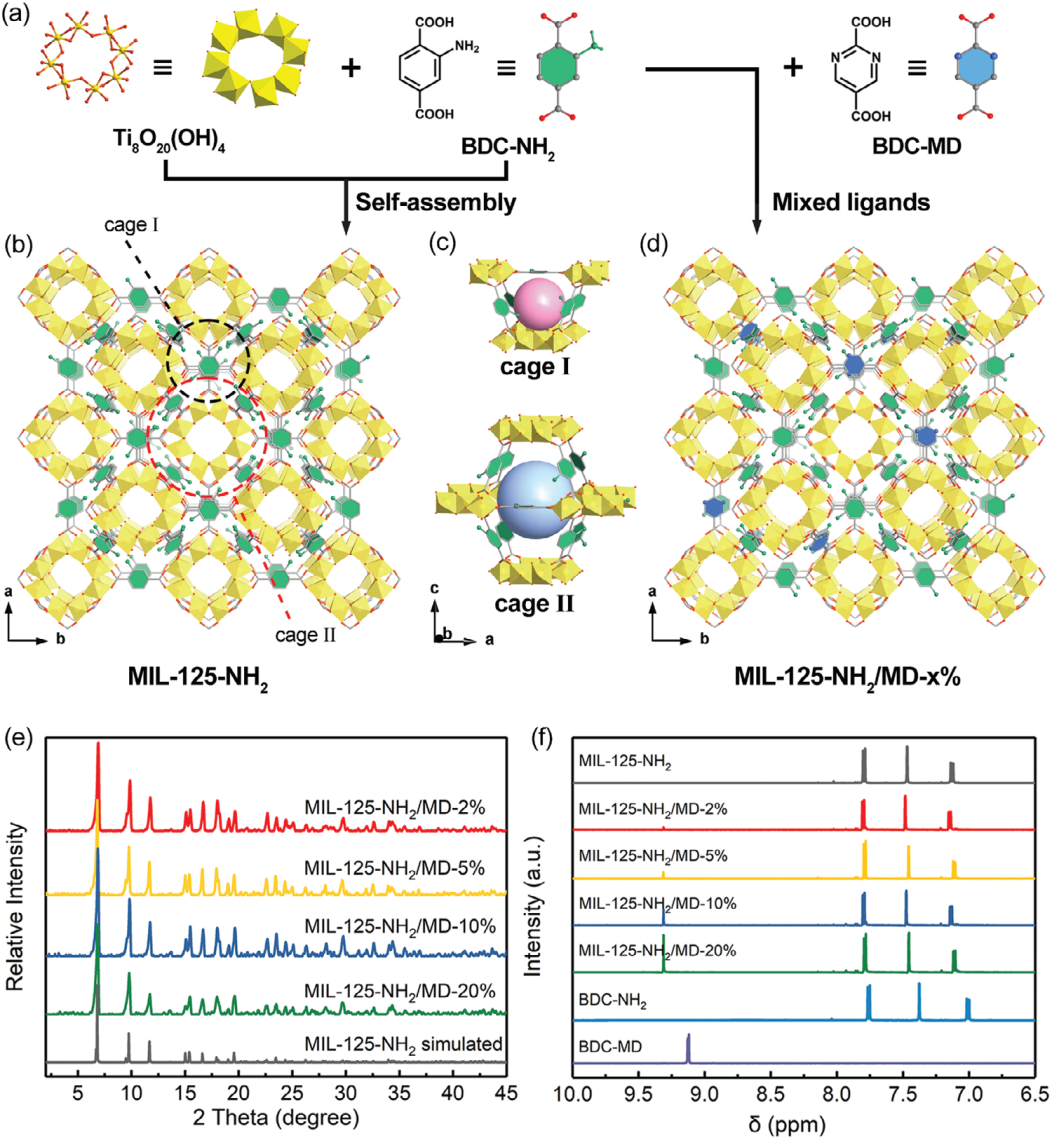
Structure characterization of MIL‐125‐NH_2_ and MIL‐125‐NH_2_/MD‐x%. a) The Ti_8_O_20_(OH)_4_ SBU and BDC‐NH_2_ ligands. b) The crystal structure of MIL‐125‐NH_2_ viewed along the *c*‐axis. c) The octahedral and tetrahedral cages in the structure of MIL‐125‐NH_2_ series. d) The BDC‐MD ligand and structure diagram of MIL‐125‐NH_2_/MD‐x%. Color code: Ti (yellow), O (red), C (grey), N (green in BDC‐NH_2_, and blue in BDC‐MD). The H atoms are omitted for clarity. e) The PXRD patterns of as‐synthesized MTV‐MOFs compared with the simulated XRD pattern from the crystal structure of MIL‐125‐NH_2_. f) ^1^H NMR spectra of MIL‐125‐NH_2_/MD‐x%, MIL‐125‐NH_2_ and the related ligands.

Recent studies have shown that the multivariate strategy of making MOFs enables broadly tunable water adsorption properties for atmospheric water harvesting and heat transfer.^[^ ^43^, ^44^, ^45^, ^46^, ^47^ ^]^ Our previous studies revealed that the immobilization of Lewis basic nitrogen sites into MOFs can enforce water binding affinity while maintaining pore volumes, thus improve low‐pressure water uptake amounts.^[^ ^48^, ^49^ ^]^ With these considerations in mind, we speculated that the use of a multivariate strategy to incorporate Lewis basic nitrogen sites into MOFs may provide a general handle for controlling the hydrophilic nature of the pores to improve low‐pressure water uptakes and thus overcome the above‐mentioned limitations. Herein, we chose a Ti‐MOF (MIL‐125‐NH_2_) as the potential platform to prove the multivariate strategy that can boost cooling performance. MIL‐125‐NH_2_ exhibits the strong water stability and high water adsorption capacity at *P*/*P*_0_ = 0.3, which is unsuitable to be used for refrig‐2 applications.^[^ ^50^, ^51^, ^52^, ^53^ ^]^ Further, direct synthesis of isomorphic Ti‐MOFs by using functional organic linkers cannot be achieved. In this context, we sought to use the post‐synthetic strategy to incorporate bare nitrogen sites into MIL‐125‐NH_2_ (**Figure** **1**), by making use of ligand exchange of 2‐aminoterephthalic acid (BDC‐NH_2_) to pyrimidine‐2,5‐dicarboxylic acid (BDC‐MD). We found that the low‐pressure water uptake and adsorption inflection point can be tunable in this series of MTV‐MOFs by controlling the content of BDC‐MD via the post‐synthetic linker exchange. Amongst this series of MTV‐MOFs, MIL‐125‐NH_2_/MD‐5% exhibits typically S‐shaped water adsorption isotherms with the best water adsorption performance. The water adsorption step is advanced from the pressure range of *P*/*P*_0_ = 0.22–0.3 in MIL‐125‐NH_2_ to *P*/*P*_0_ = 0.1–0.2 in MIL‐125‐NH_2_/MD‐5%. As a result, this material shows one of the highest water uptake capacity up to 0.39 g g^−1^ at 298 K and 20% relative humidity (RH), which has three times improvement compared with that of MIL‐125‐NH_2_ (0.12 g g^−1^). This value is even comparable to the reported benchmark materials including KMF‐1 (0.40 g g^−1^),^[^ ^41^ ^]^ and MIP‐200 (0.36 g g^−1^).^[^ ^54^ ^]^ For the standards of refrig‐2, MIL‐125‐NH_2_/MD‐5% achieves by far the record COP value (0.8) and working capacity of 0.24 g g^−1^ under an ultralow driving temperature of 65 °C, even outperforming those of some best‐performing MOFs including MIP‐200 (0.74 and 0.11 g g^−1^),^[^ ^54^ ^]^ KMF‐2 (0.62and 0.16 g g^−1^)^[^ ^43^ ^]^ and KMF‐1 (0.42 and 0.06 g g^−1^).^[^ ^41^ ^]^

## 2 Result and Discussion

### 2.1 Synthesis and Characterization

Although the water‐stable MIL‐125‐NH_2_ shows the S‐shaped water sorption isotherms with high water uptake at *P*/*P*_0_ = 0.3, its insufficient pore hydrophilicity leads to poor water uptake at *P*/*P*_0_ = 0.2 (only 0.12 g g^−1^), making it useless for refrig‐2 applications. In order to improve its hydrophilicity without sacrificing pore volume, we first attemped to construct an isoreticular MOF structure by pyrimidine‐2,5‐dicarboxylic acid (BDC‐MD) with high‐density Lewis basic nitrogen sites as a linker. We speculated that the BDC skeleton with two bare nitrogen sites in BDC‐MD can not only provide additional water binding sites but also maintain its high pore volume, making the desired material have great potential to achieve ultrahigh water uptake below *P*/*P*_0_ = 0.2. Despite extensive attempts, the synthesis of pure MIL‐125‐MD structure by using BDC‐MD as single ligand was not successful. In this context, a multivariate strategy was adopted to construct MTV‐MOFs via a post‐synthetic linker exchange method, in which we can control the mixed‐ligand ratio to fine‐tune the low‐pressure water uptake.

The high‐quality powder sample of MIL‐125‐NH_2_ was first synthesized according to the previously reported literature (Figure S2, Supporting Information).^[^ ^53^, ^55^ ^]^ Then, the multivariate strategy was conducted by partially replacing BDC‐NH_2_ with BDC‐MD through a post‐synthetic ligand exchange (more details are shown in 4. Experimental section). A series of MTV‐MOFs (named as MIL‐125‐NH_2_/MD‐x%, where x represents the BDC‐MD proportion) were designed and synthesized by incorporating different amounts of BDC‐MD ligand into MIL‐125‐NH_2_. As shown in Figure 1e, the powder X‐ray diffraction (PXRD) patterns of the as‐synthesized MIL‐125‐NH_2_/MD‐x% samples match well with the simulated pattern of MIL‐125‐NH_2_, indicating that their structures are the same with MIL‐125‐NH_2_. After that, ^1^H nuclear magnetic resonance (NMR) spectra and element analysis (EA) were used to investigate the ligand contents in these MTV‐MOFs. Prior to the measurement, all samples were carefully washed and activated to eliminate the influence from extraneous factors such as residual BDC‐MD solution or unexchanged BDC‐MD linkers in the pores. As shown in Figure 1f, the ^1^H NMR results of activated MIL‐125‐NH_2_/MD‐x% samples preserve the BDC‐NH_2_ signals (at 7.79, 7.47, and 7.13 ppm), whereas a new signal corresponding to BDC‐MD ligand appears at 9.31 ppm, revealing that the ligand exchange was successful and the BDC‐MD ligand has been incorporated. The contents of BDC‐MD (x%) in these MTV‐MOFs were calculated to be 2%, 5%, 10%, and 20%, respectively (Figure S3–S6, Supporting Information). These results were confirmed by EA measurement once again. As depicted in Figure S7 (Supporting Information), the BDC‐MD contents determined from EA tests are consistent well with that of ^1^H NMR data, which are found to be proportional to the input linker concentration. All these evidences confirm that the BDC‐MD ligand has been successfully incorporated into the MIL‐125‐NH_2_ framework with maintaining the entire structure.

As depicted in Figure 1a–d, the framework of MIL‐125‐NH_2_/MD‐x% is isostructural to MIL‐125‐NH_2_, which is self‐assembled by the Ti_8_O_20_(OH)_4_ secondary building units (SBUs) and 12 organic linkers to form a 3D *bcu* network. Such a quasi‐cubic tetragonal structure exhibits two types of pore cages (Figure 1c): one small tetrahedral cage (≈6 Å) and the other large octahedral cage (≈12 Å). The replacement of BDC‐NH_2_ by using BDC‐MD in MIL‐125‐NH_2_ may improve pore volume and hydrophilicity, since two bare N sites in BDC‐MD show more hydrophilic and less space volume compared with the amino group in BDC‐NH_2_ (Figure 1d). Therefore, the incorporation of BDC‐MD ligand into MIL‐125‐NH_2_/MD‐x% has the great potential to significantly alter the pore characteristics, thus resulting in the fine‐tuning of the low‐pressure water adsorption properties.

### 2.2 Water Adsorption Properties

**Figure 2:**
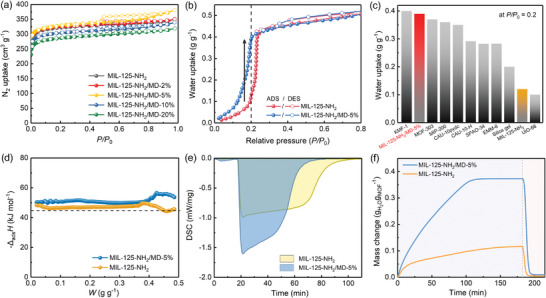
a) N_2_ sorption isotherms at 77 K of MIL‐125‐NH_2_/MD‐x% and MIL‐125‐NH_2_. b) Comparison of water adsorption isotherms of MIL‐125‐NH_2_/MD‐5% and MIL‐125‐NH_2_ at 298 K. c) Comparison of the water uptake of MIL‐125‐NH_2_/MD‐5%, MIL‐125‐NH_2_ and other previously reported adsorbents at *P*/*P*_0_ = 0.2 and room temperature. d) Isosteric heat of water adsorption (–Δ_ads_ *H*) of MIL‐125‐NH_2_/MD‐5% and MIL‐125‐NH_2_. e) Water adsorption enthalpies of MIL‐125‐NH_2_/MD‐5% and MIL‐125‐NH_2_, measured by simultaneous thermal analyzer. f) Kinetic water adsorption curves of MIL‐125‐NH_2_ and MIL‐125‐NH_2_/MD‐5%, with adsorption at 25 °C and 20% RH and subsequently desorption at 65 °C.

Nitrogen (N_2_) adsorption measurements were first conducted to investigate the pore characteristics of these MTV‐MOFs and compare them with the original MIL‐125‐NH_2_. As shown in **Figure** **2**a, all these materials exhibit typical‐I isotherm profiles, indicating their microporous characteristics (Figure S8 and Table S2, Supporting Information). MIL‐125‐NH_2_ exhibits a saturated N_2_ uptake of 349 cm^3^ g^−1^ at 77 K, affording a Brunauer−Emmett−Teller (BET) surface area of 1288 m^2^ g^−1^. These results are consistent with the values reported in the literature.^[^ ^55^ ^]^ For these MTV‐MOFs, we found that MIL‐125‐NH_2_/MD‐2% and −5% with low BDC‐MD contents show the slight increase in saturated N_2_ uptake and surface area (350 cm^3^ g^−1^ and 1303 m^2^ g^−1^, 380 cm^3^ g^−1^ and 1315 m^2^ g^−1^ for MIL‐125‐NH_2_/MD‐2% and −5%, respectively). This is because two N sites in BDC‐MD show less space volume compared with the amino group in BDC‐NH_2_, leading to the slightly enhanced surface areas for MTV‐MOFs. However, with the loading amount increased, MIL‐125‐NH_2_/MD‐10% and −20% show a slightly decreased N_2_ uptakes and surface areas, which might because the high linker exchange led to the loss of high crystallinity. Therefore, MIL‐125‐NH_2_/MD‐5% shows the potential to achieve the best low‐pressure water uptakes, due to its most balance between high BET surface area and high density of hydrophilic N sites among these MTV‐MOFs.

Water adsorption isotherms of MIL‐125‐NH_2_ and these MTV‐MOFs were systematically measured at different temperatures (Figure 2b; Figure S9–S12, Supporting Information). As shown in Figure S9 (Supporting Information), all these MOFs show typical S‐shaped adsorption isotherms under 298 K, showing different step adsorption pressures and water uptakes that can be tuned by changing the ligands. First, we discovered that the incorporation of small amounts of BDC‐MD linker (e.g., 2% and 5%) have a negligible influence on the saturated water uptake. However, the further increase of BDC‐MD amounts in MIL‐125‐NH_2_/MD‐10% would lead to the decrease in the total water uptake due to the decreased surface area, which is detrimental to improve water uptake properties. More importantly, all these MTV‐MOFs can shift the water adsorption inflection point towards lower pressure below *P*/*P*_0_ = 0.2 due to the more hydrophilia from the BDC‐MD ligand (Figure S9, Supporting Information), which can be tuned by changing the ligand contents. Owing to the combination of high surface area, crystallinity and high density of Lewis N sites, MIL‐125‐NH_2_/MD‐5% shows the best water uptake behavior in low‐pressure regions amongst these MTV‐MOFs. As shown in Figure 2b, the step adsorption pressure was decreased from *P*/*P*_0_ = 0.23 in MIL‐125‐NH_2_ to *P*/*P*_0_ = 0.18 in MIL‐125‐NH_2_/MD‐5%. Such notably increased pore hydrophilicity results in significantly enhanced low‐pressure water uptakes, wherein MIL‐125‐NH_2_/MD‐5% shows a 3.2 times higher water uptake at *P*/*P*_0_ = 0.2 (0.39 g g^−1^) than that of the pristine MIL‐125‐NH_2_ (0.12 g g^−1^). It is worthy to note that this value is much higher than most of the top‐performing materials such as CAU‐10 (0.29 g g^−1^)^[^ ^34^ ^]^ and MIP‐200 (0.36 g g^−1^),^[^ ^54^ ^]^ and even comparable to that of the benchmark KMF‐1 (0.40 g g^−1^)^[^ ^41^ ^]^ (Figure 2c). These observations on the improvement of water uptake at *P*/*P*_0_ = 0.2 for MIL‐125‐NH_2_/MD‐5% enable us to maximize the working capacity and thus improve the performance for refrig‐2 cooling applications.

These changes in low‐pressure water adsorption of MIL‐125‐NH_2_/MD‐5% can be explained by the isosteric heat of water adsorption (−Δ_ads_ *H*). Based on water adsorption isotherms collected at different temperatures, the −Δ_ads_ *H* values of MIL‐125‐NH_2_/MD‐5% and MIL‐125‐NH_2_ were calculated by the Clausius–Clapeyron equation (Figure S11 and S12, Supporting Information). As shown in Figure 2d, the −Δ_ads_ *H* values of MIL‐125‐NH_2_/MD‐5% are higher than those of MIL‐125‐NH_2_ within the whole adsorption stage, revealing its better hydrophilicity and water binding affinity. The average value of −Δ_ads_ *H* for MIL‐125‐NH_2_/MD‐5% is moderate (50.7 kJ mol^−1^) and only slightly higher than the evaporation enthalpy of water (44.19 kJ mol^−1^), thus showing the potential to be regenerated under low desorption temperatures. These results were further confirmed by applying an experimental method on a simultaneous thermal analyzer. As can be seen from Figure 2e, the differential scanning calorimetry (DSC) curve of MIL‐125‐NH_2_/MD‐5% exhibits a larger exothermic peak than that of MIL‐125‐NH_2_, which also indicates a higher water binding affinity for MIL‐125‐NH_2_/MD‐5%. These observations from both theoretical and experimental results demonstrated that the incorporation of BDC‐MD ligand into MTV‐MOFs can enhance the water binding affinity, thus resulting in the step pressure shifted to lower pressures and the highly enhanced water uptake at *P*/*P*_0_ = 0.2.

Kinetic sorption behaviors of water adsorbents are another key factor to determine the practical applications, since the water adsorption/desorption cycle time directly impacts the heat transfer efficiency. Prior to adsorption measurements, the powder samples of MIL‐125‐NH_2_ and MIL‐125‐NH_2_/MD‐5% were fully activated and then measured with the operating conditions between adsorption at 25 °C/20% RH and desorption at 65 °C/0% RH. As shown in Figure 2f, the insufficient hydrophilicity of MIL‐125‐NH_2_ caused a very slow kinetic adsorption behavior at 20% RH and 25 °C, requiring more than 180 min to reach an adsorption saturation. However, the resulting MIL‐125‐NH_2_/MD‐5% exhibits faster kinetic water adsorption, with the saturation time shortened to 120 min, indicating its notably improved pore hydrophilicity and adsorption kinetics. Whereafter, the adsorbed water in MIL‐125‐NH_2_/MD‐5% can be fully desorbed within 20 min at 65 °C, verifying its exceptional ability for the ultralow‐temperature desorption. Therefore, through a simple multivariate strategy, the designed MTV‐MOF, MIL‐125‐NH_2_/MD‐5%, overcomes the low‐pressure water adsorption limitations of pristine MIL‐125‐NH_2_, resulting in much higher water uptake (0.39 g g^−1^) at *P*/*P*_0_ = 0.2 and faster water adsorption/desorption kinetics. These comprehensive advantages make MIL‐125‐NH_2_/MD‐5% greatly meet the prerequisites for achieving high refrig‐2 cooling performance.

### 2.3 AC Performance Assessments

**Figure 3:**
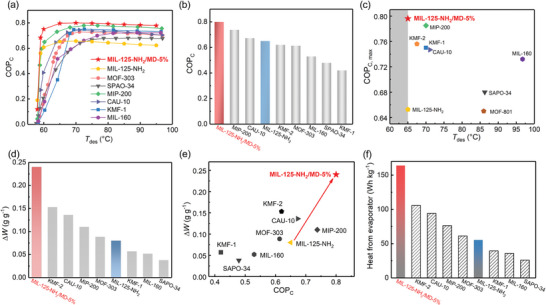
a) COP_C_ plots for AC conditions (*T*_ev_ = 5 °C, *T*_con_ = 30 °C) as a function of desorption temperature (*T*_des_). b) Comparison of COP_C_ of MIL‐125‐NH_2_/MD‐5% and other benchmark materials, examined under standard AC conditions (*T*_ev_ = 5 °C, *T*_con_ = 30 °C, *T*_ads_ = 30 °C, and *T*_des_ = 65 °C). c) Maximum COP_C_ value and their corresponding driven temperature for the indicated materials, compared under *T*_ev_ = 5 °C, *T*_con_ = 30 °C. d) The gravimetric working capacity (Δ*W*) of MIL‐125‐NH_2_/MD‐5% and other benchmark materials, compared at *T*_ev_ = 5 °C, *T*_con_ = 30 °C, *T*_ads_ = 30 °C, and *T*_des_ = 65 °C. e) COP_C_ versus gravimetric working capacity (Δ*W*), examined under standard AC conditions (*T*_ev_ = 5 °C, *T*_con_ = 30 °C, *T*_ads_ = 30 °C, and *T*_des_ = 65 °C). f) Heat from evaporator for MIL‐125‐NH_2_/MD‐5% and some promising adsorbents expressed in gravimetry scales. Boundary conditions: heats transferred from the evaporator in one cooling cycle at *T*_ev_ = 5 °C, *T*_ads_ = 30 °C, *T*_con_ = 30 °C, and *T*_des_ = 65 °C.

The excellent water sorption properties of MIL‐125‐NH_2_/MD‐5% inspired us to evaluate its thermodynamic performance for refrig‐2 applications at ultralow driving temperatures. The working efficiencies of water adsorbents in refrig‐2 applications can be assessed by COP for cooling (COP_C_), which was determined by thermodynamic models applied at standard boundary temperature conditions for water evaporation (*T*_ev_), condensation (*T*_con_), adsorption (*T*_ads_), and desorption/regeneration (*T*_des_).^[^ ^9^ ^]^ For better understanding, a schematic diagram of the thermodynamic cycle between MOF and water is presented in Figure S13 (Supporting Information), and the details of standard boundary temperature conditions at different cooling applications are shown in Table S1 (Supporting Information).^[^ ^9^ ^]^ The characteristic curves of MIL‐125‐NH_2_ and MIL‐125‐NH_2_/MD‐5% were first transformed from their water adsorption isotherms (Figure S14 and S15, Supporting Information), and the COP_C_ values were calculated according to the well‐established methodology under the given standard refrigeration conditions for refrig‐2 (*T*_ev_ = 5 °C and *T*_con_ = 30 °C).^[^ ^9^, ^10^ ^]^ As depicted in **Figure** **3**a, with the *T*_des_ increased, the COP_C_ value of MIL‐125‐NH_2_/MD‐5% rises gradually below 60 °C and then reaches maximum at 65 °C, indicating that MIL‐125‐NH_2_/MD‐5% has the potential to realize ultralow‐temperature‐driven ACs. At ultralow driving temperatures of 60 and 65 °C, MIL‐125‐NH_2_/MD‐5% exhibits an extremely high COP_C_ value of 0.75 and 0.8, respectively, much higher than that of the pristine MIL‐125‐NH_2_ (0.61 and 0.65). These COP_C_ values also outperform most of the promising MOFs reported (Figure 3b; Figure S16, Supporting Information), such as MIP‐200 (0.64 and 0.74),^[^ ^54^ ^]^ CAU‐10 (0.42 and 0.67)^[^ ^34^ ^]^ and MOF‐303 (0.20 and 0.61),^[^ ^42^ ^]^ revealing its benchmark cooling efficiency. More importantly, the required driving temperature to achieve the maximum COP_C_ for MIL‐125‐NH_2_/MD‐5% (0.8, 65 °C) is lower than those of the best‐performing materials at least 5 °C (Figure 3c), like MIP‐200 (0.78, 70 °C),^[^ ^54^ ^]^ KFM‐1 (0.75, 70 °C),^[^ ^41^ ^]^ KMF‐2 (0.76, 67.5 °C)^[^ ^43^ ^]^ and CAU‐10 (0.75, 71 °C),^[^ ^34^ ^]^ which is significant for the efficient utilization of ultra‐low‐grade thermal energy.

In addition to the COP_C_, the water working capacity (Δ*W*) between the adsorption and desorption stages is also very important because it determines the amount of energy that can be transferred from the evaporator. When setting *T*_des_ = 65 °C, the gravimetric Δ*W* of MIL‐125‐NH_2_/MD‐5% is up to 0.24 g g^−1^, far surpassing the 0.08 g g^−1^ of MIL‐125‐NH_2_ (Figure 3d; Figure S17, Supporting Information). Such a large improvement is caused by its highly enhanced water uptake at *P*/*P*_0_ = 0.2. It is worthy of noting that this working capacity of 0.24 g g^−1^ is the highest reported so far at *T*_des_ = 65 °C, far exceeding most of advanced adsorbents like KMF‐2 (0.16 g g^−1^),^[^ ^43^ ^]^ CAU‐10 (0.14 g g^−1^),^[^ ^34^ ^]^ MIP‐200 (0.11 g g^−1^),^[^ ^54^ ^]^ and MOF‐303 (0.09 g g^−1^),^[^ ^42^ ^]^ showing its great advantage in transferring energy under such ultralow driving temperature. Considering both the COP_C_ and Δ*W* values play the important roles in determining the overall system efficiency, we thus put them together as concurrent objectives and compared with the indicated top‐performing materials. As depicted in Figure 3e, MIL‐125‐NH_2_/MD‐5% exhibits by far both the highest COP_C_ and working capacity under an ultralow driving temperature of 65 °C, making it as the benchmark for achieving highly efficient cooling under refrig‐2 conditions.

Considering the superior cooling performance of MIL‐125‐NH_2_/MD‐5%, the gravimetric heat value from the evaporator was estimated and then compared with other promising adsorbents under specific working conditions. As shown in Figure 3f, MIL‐125‐NH_2_/MD‐5% exhibits an excellent value of 165 Wh kg^−1^ for the evaporative storage capacity in a single refrigeration cycle (at *T*_ev_ = 5 °C, *T*_ads_ = 30 °C, and *T*_des_ = 65 °C), which is three times higher than the original MIL‐125‐NH_2_ (53.9 Wh kg^−1^), and far exceeds that of KMF‐2 (105.8 Wh kg^−1^),^[^ ^43^ ^]^ CAU‐10 (94.2 Wh kg^−1^),^[^ ^34^ ^]^ MIP‐200 (76.2 Wh kg^−1^),^[^ ^54^ ^]^ and MOF‐303 (61.2 Wh kg^−1^).^[^ ^42^ ^]^ Moreover, the storage capacity of MIL‐125‐NH_2_/MD‐5% can be further increased to 222.6 Wh kg^−1^ at a higher regeneration temperature (*T*_des_ = 70 °C), as shown in Table S3 (Supporting Information). These results make this material among the best water adsorbents for refrig‐2 applications.

### 2.4 Stability Measurements

**Figure 4:**
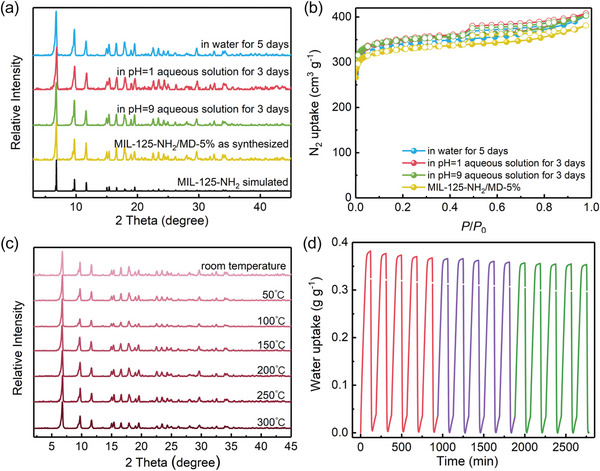
a) PXRD patterns and b) 77 K N_2_ adsorption isotherms of MIL‐125‐NH_2_/MD‐5% samples after treatment with different conditions. c) Variable‐temperature PXRD patterns for MIL‐125‐NH_2_/MD‐5%. d) Water adsorption/desorption cycling performance of MIL‐125‐NH_2_/MD‐5%.

Besides high cooling performances, excellent water and cycling stabilities are also required for water adsorbents to undergo the hash working conditions in actual applications. The chemical stability of MIL‐125‐NH_2_/MD‐5% was first examined, monitored by the PXRD, scanning electron microscopy (SEM), and N_2_ adsorption isotherms. Before measurements, the fresh samples of MIL‐125‐NH_2_/MD‐5% were immersed in water for 5 days, and in aqueous solutions of pH = 1 and = 9 for 3 days. As shown in **Figure** **4**a, the PXRD profiles demonstrated that MIL‐125‐NH_2_/MD‐5% can maintain its structural integrity without any phase change or loss of crystallinity after treatment in different harsh conditions. The SEM images of treated crystals existed an unchangeable surface and intact round‐shape morphology (Figure S18, Supporting Information). This robust nature of MIL‐125‐NH_2_/MD‐5% was further confirmed by N_2_ adsorption measurements at 77 K, wherein these N_2_ adsorption isotherms almost coincided with those of the pristine sample (Figure 4b). Next, we investigated its thermal stability by variable temperature PXRD patterns, thermogravimetric curves, and N_2_ adsorption isotherms, which showed that MIL‐125‐NH_2_/MD‐5% is thermally stable up to 300 °C (Figure 4c; Figure S19 and S20, Supporting Information). Then, the cycling stability of MIL‐125‐NH_2_/MD‐5% was evaluated using the gravimetric water‐sorption cycles. As shown in Figure 4d; Figure S21 (Supporting Information), MIL‐125‐NH_2_/MD‐5% maintains a steady water uptake of 0.37 g g^−1^ after 15 consecutive cycles and no changes on PXRD profiles were observed, thus proving its high cycling durability. Therefore, MIL‐125‐NH_2_/MD‐5% shows one of the best chemical, thermal, and cycling stabilities among the reported MOFs.

## 3 Conclusion

In conclusion, we have proposed and demonstrated that a multivariate strategy of incorporating Lewis basic nitrogen sites into MOFs can improve the low‐pressure water uptake, and thus highly boost their COP_C_ and Δ*W* for cooling applications. By elaborately altering the contents of the second organic ligand, the resulting MTV‐MOFs showed highly tunable and enhanced hydrophilicity due to the incorporation of bare nitrogen sites derived from BDC‐MD. The best MIL‐125‐NH_2_/MD‐5% thus exhibits typically S‐shaped adsorption isotherms with an ultrahigh water uptake of 0.39 g g^−1^ at *P*/*P*_0_ = 0.2 and 298 K, which is more than three times higher than the pristine MIL‐125‐NH_2_ (0.12 g g^−1^) and is even comparable with the benchmark materials like MIP‐200 (0.36 g g^−1^) and KMF‐1 (0.40 g g^−1^). Such an enhanced low‐pressure water uptake resulted in a significant improvement on MIL‐125‐NH_2_/MD‐5% for both COP_C_ and Δ*W* under refrig‐2 conditions, with one of the highest values of 0.8 and 0.24 g g^−1^ reported so far under the ultralow driving temperature of 65 °C. These values are notably higher than the pristine MIL‐125‐NH_2_ (0.65 and 0.08 g g^−1^) and most of the best‐performing materials reported such as MIP‐200 (0.74 and 0.11 g g^−1^) and KMF‐2 (0.62 and 0.16 g g^−1^). Combined with its enhanced water sorption kinetics and high cycling durability, MIL‐125‐NH_2_/MD‐5% is placed among one of the most promising adsorbents for refrig‐2 applications. This work may provide a powerful strategy to functionalize and modulate the hydrophilicity of MOFs to improve the low‐pressure water uptake for targeting high‐performance AC applications.

## 4 Experimental Section

### Materials and Methods

All reagents were purchased from commercial sources and used without further purification. 2‐aminoterephthalic acid and pyrimidine‐2,5‐dicarboxylic acid were purchased from Bidepharm. Dried methanol and dried N,N‐dimethylformamide were purchased from Energy Chemical. And, tetrabutyl titanate was purchased from Aladdin. ^1^H nuclear magnetic resonance (NMR) spectra were recorded on JNM‐ECZ500R/M1 nuclear magnetic resonance spectrometer (500 MHz). Powder X‐ray diffraction (PXRD) patterns were collected on an X'Pert PRO diffractometer with Cu‐K*α* (*λ* = 1.542 Å) radiation, and scanned at a rate of 5° min^−1^ in the range of 2–45° (2𝜃) under ambient conditions. Element analysis (EA) was measured by Elementar Unicube elemental analyzer. Scanning electron microscope (SEM) pictures were performed by Hitachi S‐4800 field emission SEM. Thermogravimetric analyses (TGA) were developed on a TA SDT 650 thermal analyzer under nitrogen atmosphere. Differential Scanning Calorimetry (DSC) was performed by Netzsch STA 449F3 simultaneous thermal analyzer.

### Synthesis of MIL‐125‐NH_2_ and MIL‐125‐NH_2_/MD‐x%

MIL‐125‐NH_2_ was synthesized through solvothermal reactions according to the previous literature.^[^ ^53^, ^55^ ^]^ BDC‐NH_2_ (1.086 g, 6 mmol) was completely dissolved in the mixture of DMF (3.5 mL) and MeOH (3.5 mL) by sonication for about 20 min. Tetrabutyl titanate (0.51 mL, 1.5 mmol) was added to the solution and sonicated for another 5 min. Then, the resulting solution was transferred to a Teflon‐lined autoclave and placed into an oven (423 K) for 16 h. After cooling down, the crystalline powder MIL‐125‐NH_2_ was filtered and washed thoroughly with DMF until the liquid was clear. And then, the fresh sample was soaked in dry methanol for solvent‐exchange. The obtained powder was dried under vacuum at room temperature and 373 K for 12 h.

MIL‐125‐NH_2_/MD‐x% was synthesized according to previous literature with a slight modification.^[^ ^56^ ^]^ Typically for MIL‐125‐NH_2_/MD‐5%, MIL‐125‐NH_2_ (35 mg, 0.021 mmol) and BDC‐MD (33.6 mg, 0.2 mmol) were dissolved in the mixture of DMF (9 mL) and MeOH (1 mL). The uniform mixture was formed by ultrasonic treatment and transferred to a preheated oven at 423 K for 24 h. Afterward, the precipitate was isolated by filtration and washed with DMF and MeOH thoroughly. The MIL‐125‐NH_2_/MD‐2%, MIL‐125‐NH_2_/MD‐10% and MIL‐125‐NH_2_/MD‐20% were obtained by similar procedures with different amount of BDC‐MD, namely 16.8 mg/0.1 mmol, 50.4 mg/0.3 mmol and 67.2 mg/0.4 mmol, respectively.

### Gas Sorption Measurements

Before all gas sorption analyses, the fresh sample needed to be solvent‐exchanged with dry ethanol at least eight times within three days. Then, it was evacuated for 12 h at room temperature and further at 423 K for 12 h until the outgas rate was smaller than 5 µmHg min^−1^. N_2_ sorption isotherms were measured by the Micromeritics ASAP 2460 surface area analyzer and the measurement was maintained at 77 K with liquid nitrogen. Volumetric water sorption isotherms were measured by the BELSORP‐max instrument (BeL‐Japan). And the temperature was maintained at a constant temperature with a recirculating chiller.

## Conflict of Interest

The authors declare no conflict of interest.

## Supporting information

Supporting Information

## Data Availability

The data that support the findings of this study are available from the corresponding author upon reasonable request.
