# Supplementary material for: Tunable Low‐Pressure Water Adsorption in Stable Multivariate Metal‐Organic Frameworks for Boosting Water‐Based Ultralow‐Temperature‐Driven Refrigeration
Source: Adv Sci (Weinh). 2024 Jan 15;11(11):2308623. doi: 10.1002/advs.202308623 (PMC10953545; doi:10.1002/advs.202308623)
Supplement: Supplementary file 1 — Supporting Information [file ADVS-11-2308623-s001.pdf]

ADVANCED SCIENCE
Open Access

Supporting Information

for *Adv. Sci.*, DOI 10.1002/advs.202308623

Tunable Low-Pressure Water Adsorption in Stable Multivariate Metal-Organic Frameworks for Boosting Water-Based Ultralow-Temperature-Driven Refrigeration

Chen-Han Guo, Feng-Fan Lu, Enyu Wu, Jia-Xin Wang, Defa Gu, Bin Li and Guodong Qian**

Supporting information

Tunable Low-Pressure Water Adsorption in Stable Multivariate Metal–Organic Frameworks for Boosting Water-Based Ultralow-Temperature-Driven Refrigeration

Chen-Han Guo[+], Feng-Fan Lu[+], Enyu Wu, Jia-Xin Wang, Defa Gu, Bin Li, and Guodong Qian**

C.-H. Guo, F.-F. Lu, E. Wu, Dr. J.-X. Wang, Dr. D. Gu, Prof. B. Li, Prof. G. Qian
State Key Laboratory of Silicon and Advanced Semiconductor Materials, School of Materials Science and Engineering, Zhejiang University, Hangzhou 310027, China.
*E-mail: bin.li@zju.edu.cn; gdqian@zju.edu.cn
[+] These authors contributed equally to this work.

Table S1. Operational tempratures for three different kinds of refrigeration.[1]

	Refrig-1	Refrig-2	Refrig-3 (ice making)[a)]
T_{ev}/K	283	278	268
T_{con}/K	303	303	298

[a)] Water adsorbent cannot be used as working fluid for refrig-3, due to freezing.

Table S2. BET surface area and pore volume of MIL-125-NH_2, MIL-125-NH_2/MD-2%, MIL-125-NH_2/MD-5%, MIL-125-NH_2/MD-10% and MIL-125-NH_2/MD-20%.

Materials	BET surface area (m^2 g^{-1})	Pore volume (cm^3 g^{-1})
MIL-125-NH_2	1288	0.543
MIL-125-NH_2/MD-2%	1303	0.542
MIL-125-NH_2/MD-5%	1315	0.590
MIL-125-NH_2/MD-10%	1277	0.526
MIL-125-NH_2/MD-20%	1143	0.494

Table S3. Water sorption properties and specific energy capacity of MIL-125-NH_2/MD-5% and other

benchmark materials.

Materials	Working capacity[a)] (g g^{-1})	Heat from evaporator[b)] (Wh kg^{-1})	Energy storage capacity[c)] (Wh kg^{-1})	-$\Delta_{ads}H$[d)] (kJ mol^{-1})	COP_C [e)]	Ref.
MIL-125-NH_2	0.08	53.9	287	47.8	0.65	This work
**MIL-125-NH_2/MD-5%**	**0.24**	**165 (222.6)**	**290**	**50.8**	**0.8**	**This work**
MIP-200	0.11	76.2	189	51.20	0.74	[3]
KMF-1	0.06	39.5	323	52–57	0.42	[4]
MIL-160	0.05	35.8	108	50.64	0.53	[5]
CAU-10	0.14	94.2	238	53.5	0.67	[6,7]
MOF-303	0.09	61.2	255	52	0.61	[8]
KMF-2	0.16	105.8	286	55	0.62	[9]

a) Working capacity deduced from water-sorption cycles for AC conditions: T_{ev} = 5 °C, T_{ads} = 30 °C, and T_{con} = 30 °C at T_{des} = 65 °C.

b) Heat transferred from the evaporator in one refrigeration cycle at T_{ev} = 5 °C, T_{ads} = 30 °C and T_{des} = 65 °C, (T_{des} = 70 °C for values in the parenthesis).

c) Energy storage capacity per unit weight of adsorbent at T_{ev} = 10 °C, T_{ads} = 30 °C and T_{des} = 70 °C.

d) Heat of adsorption is calculated from Clausius–Clapeyron equation.

e) At T_{ev} = 5 °C, T_{ads} = 30 °C and T_{des} = 65 °C.

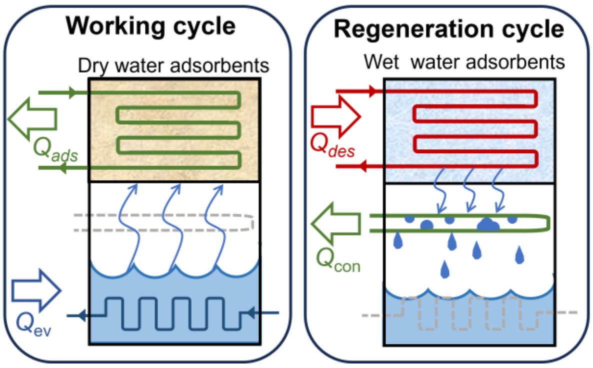

Figure S1. Schematic diagram of working cycle (left) and regeneration cycle (right) in a water-based adsorption refrigeration. Reproduced with permission.[2] Copyright 2013, Royal Society of Chemistry.

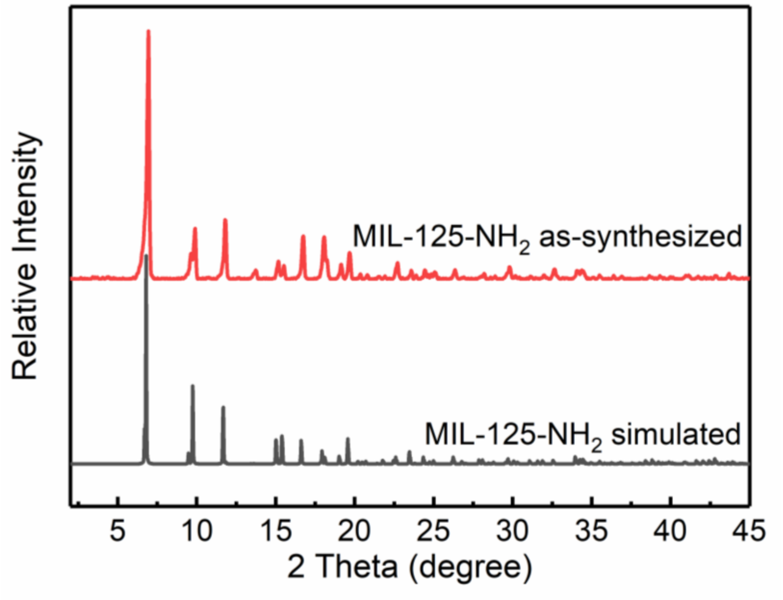

Figure S2. The PXRD patterns of as-synthesized MIL-125-NH_2, compared with the simulated XRD pattern from the structure of MIL-125-NH_2.

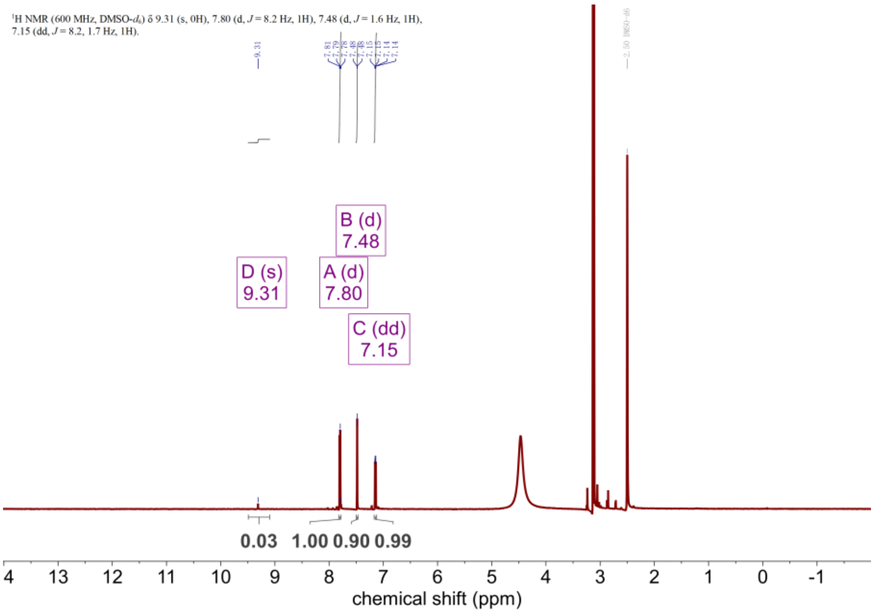

Figure S3. ^{1}H NMR (DMSO-d6, 500 MHz) spectra of MIL-125-NH_2/MD-2%.

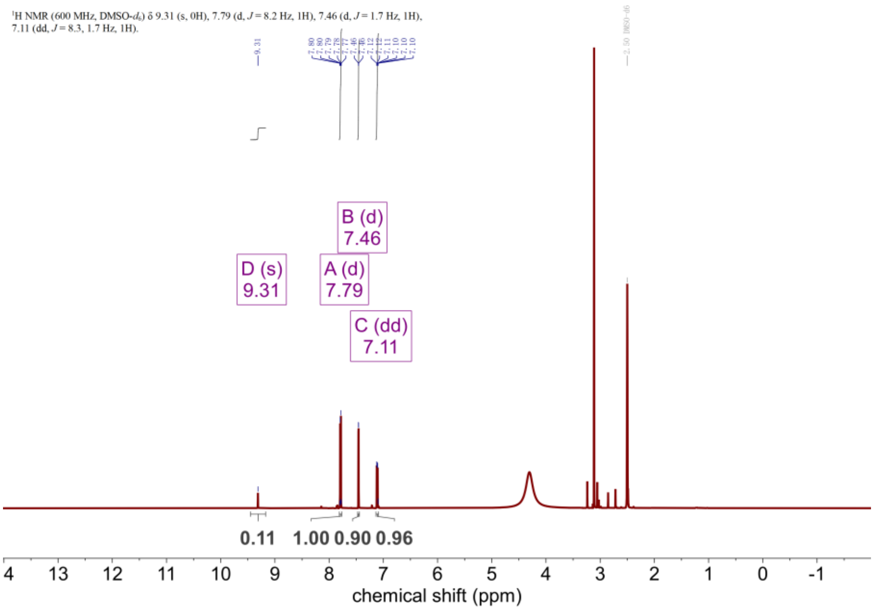

Figure S4. ^{1}H NMR (DMSO-d6, 500 MHz) spectra of MIL-125-NH_2/MD-5%.

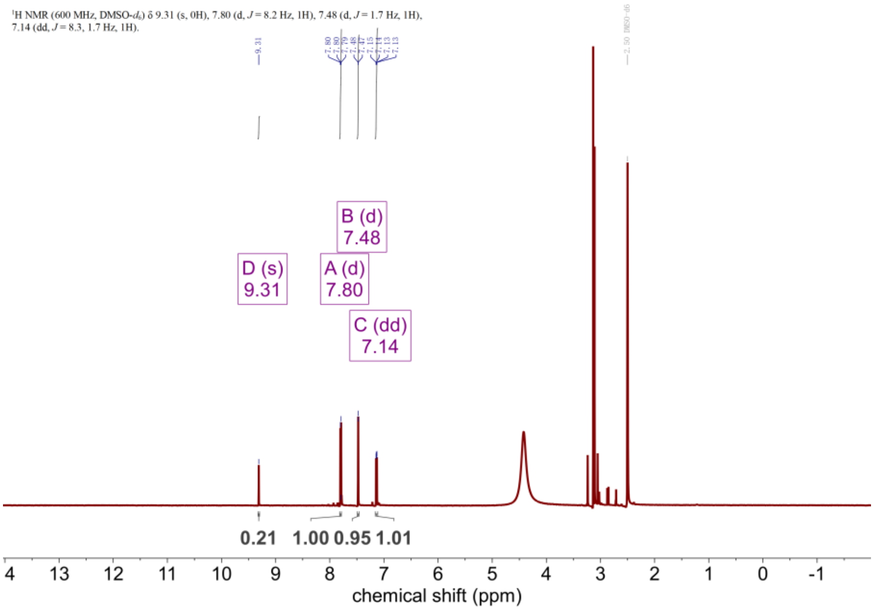

Figure S5. ^{1}H NMR (DMSO-d6, 500 MHz) spectra of MIL-125-NH_2/MD-10%.

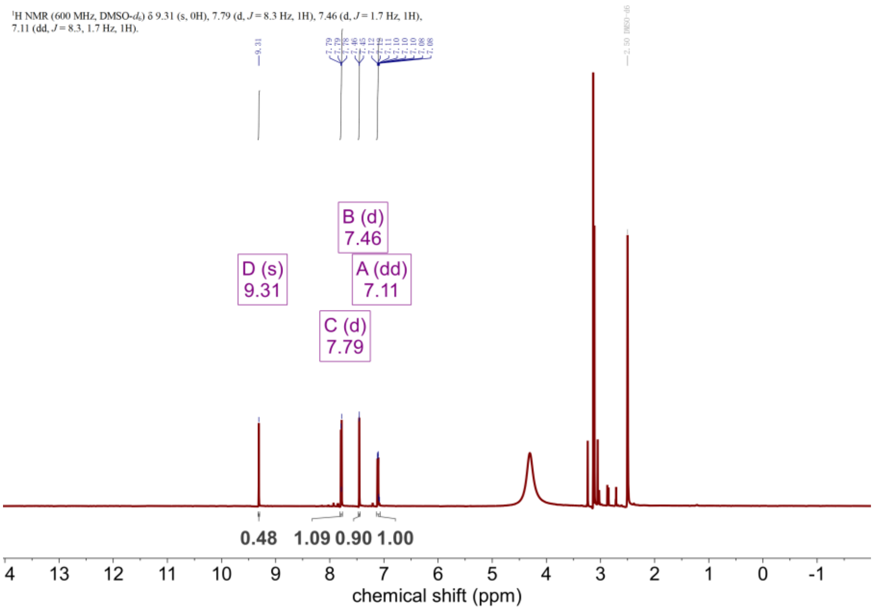

Figure S6. ^{1}H NMR (DMSO-d6, 500 MHz) spectra of MIL-125-NH_2/MD-20%.

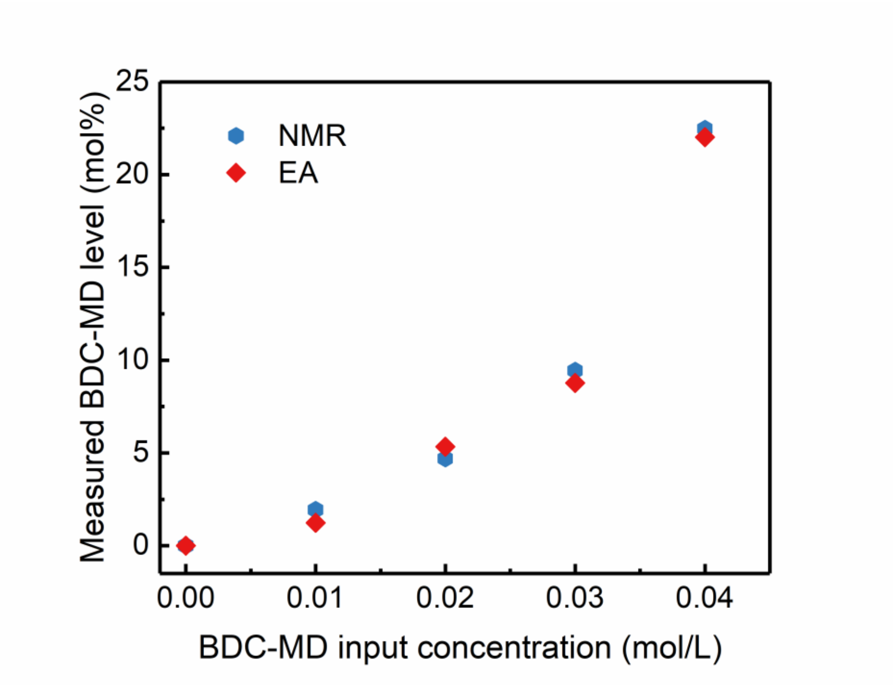

Figure S7. Molar ratios of BDC-MD linker in MIL-125-NH_2/MD-x% determined by NMR and EA.

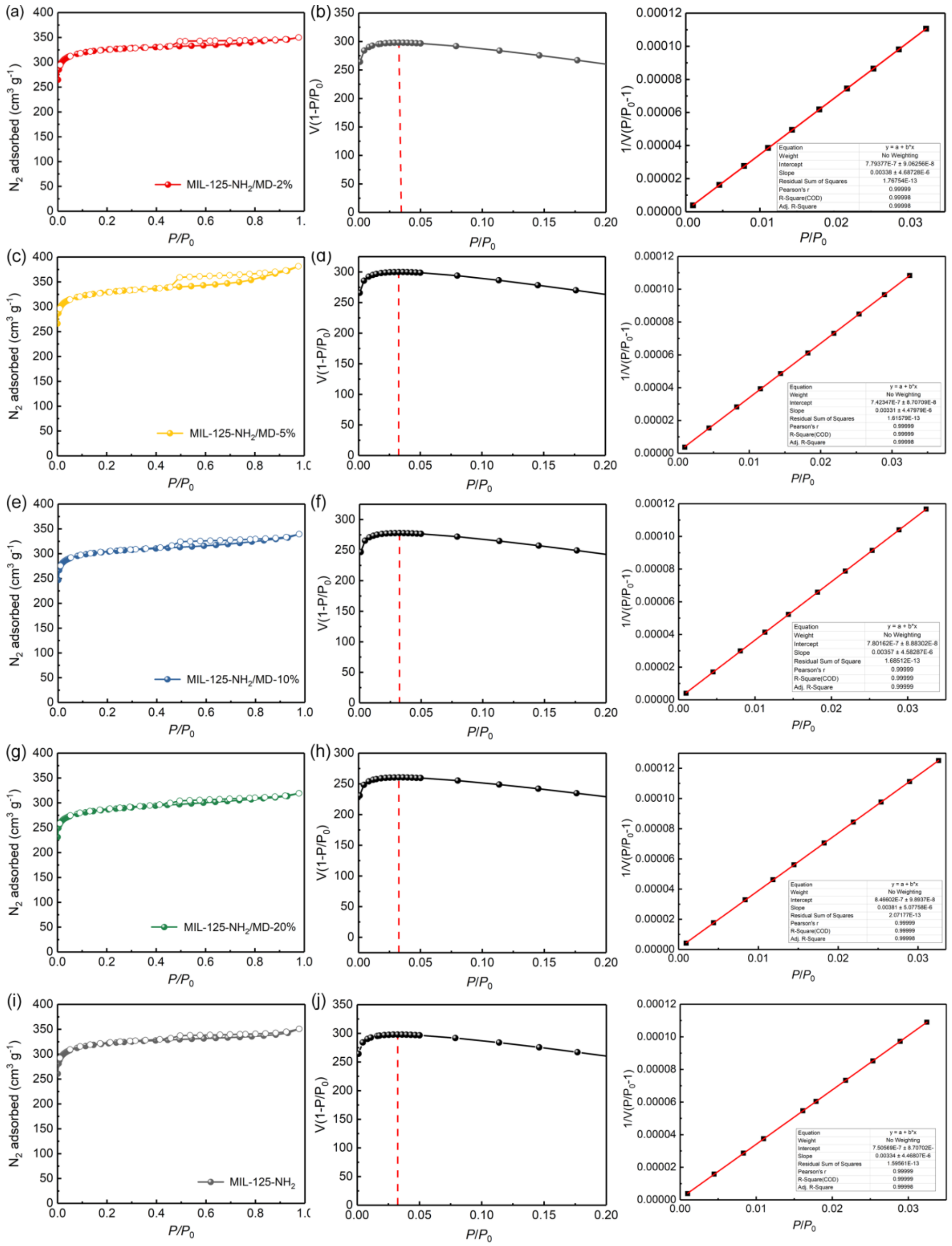

Figure S8. N_2 adsorption isotherms at 77 K of MIL-125-NH_2/MD-2% (a), MIL-125-NH_2/MD-5% (c), MIL-125-NH_2/MD-10% (e), MIL-125-NH_2/MD-20% (g) and MIL-125-NH_2 (i). BET specific surface area fitting of MIL-125-NH_2/MD-2% (b), MIL-125-NH_2/MD-5% (d), MIL-125-NH_2/MD-10% (f), MIL-125-NH_2/MD-20% (h) and MIL-125-NH_2 (j).

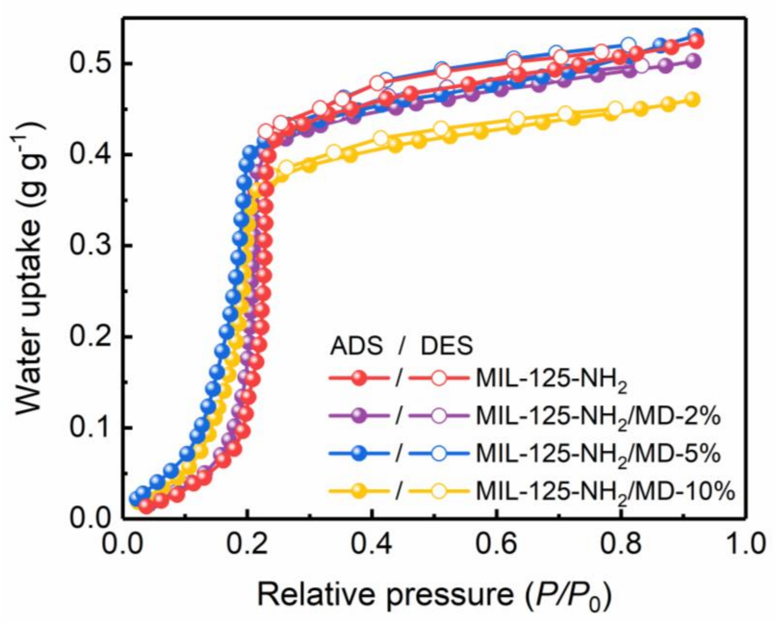

Figure S9. Water adsorption isotherms of MIL-125-NH_2/MD-2%, MIL-125-NH_2/MD-5%, MIL-125-NH_2/MD-10% and MIL-125-NH_2 at room temperature.

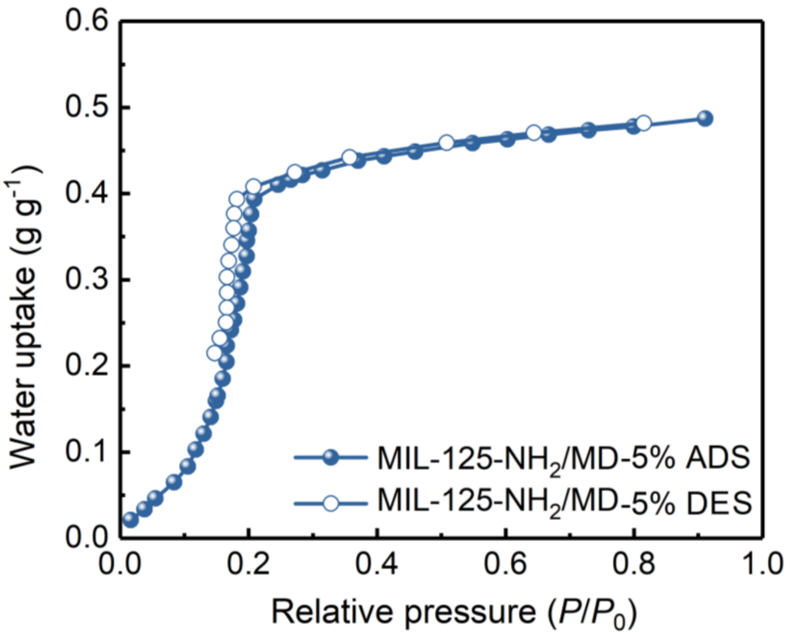

Figure S10. Water adsorption isotherms of MIL-125-NH_2/MD-5% at 298 K.

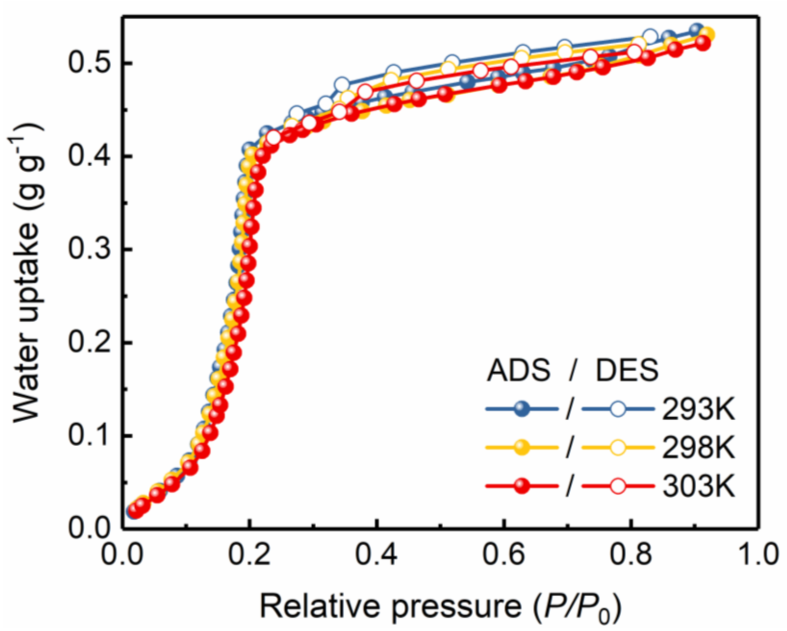

Figure S11. Water adsorption isotherms of MIL-125-NH_2/MD-5% at different temperatures under relative pressure.

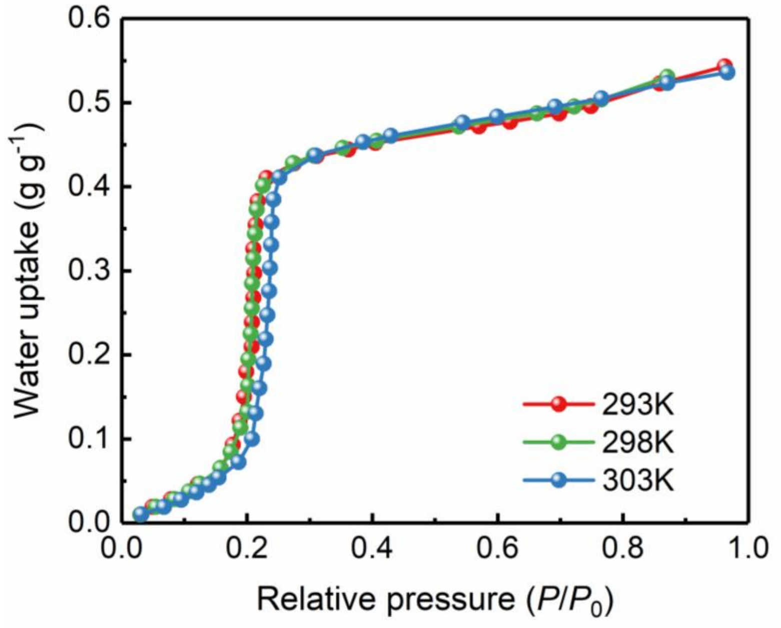

Figure S12. Water adsorption isotherms of MIL-125-NH_2 at different temperatures under relative pressure.

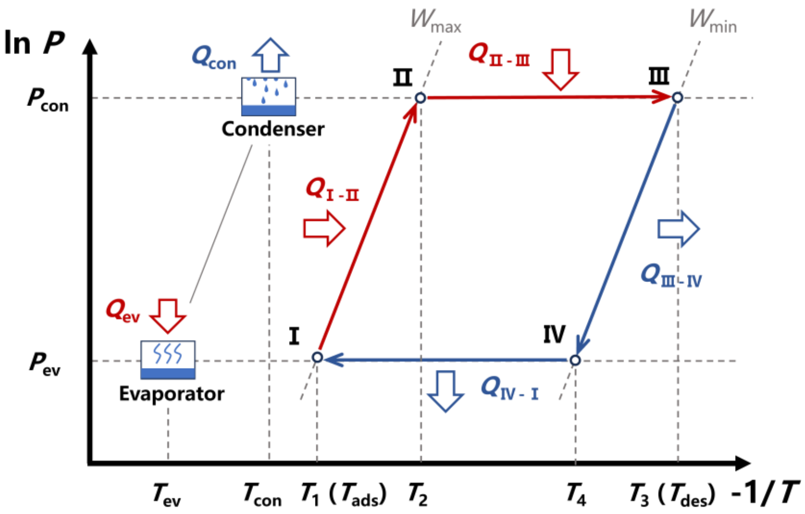

Figure S13. Schematic diagram of the thermodynamic cycle of an adsorption chiller. Reproduced with permission.[1] Copyright 2015, American Chemical Society.

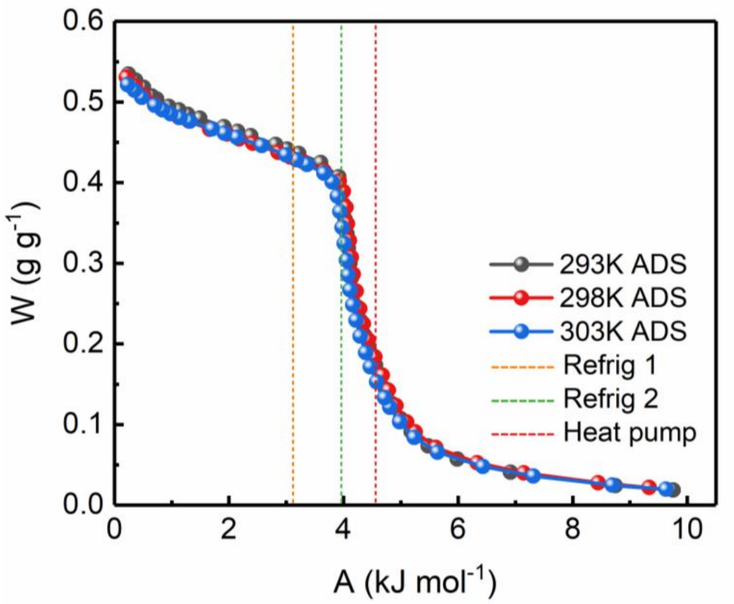

Figure S14. Characteristic curves of MIL-125-NH_2/MD-5% at different temperatures determined using eq 1 and 2. The orange dotted line represents the optimal adsorption potential for refrig-1 cooling, the green dotted line for refrig-2 cooling, and the red dotted line for heat pump heating.

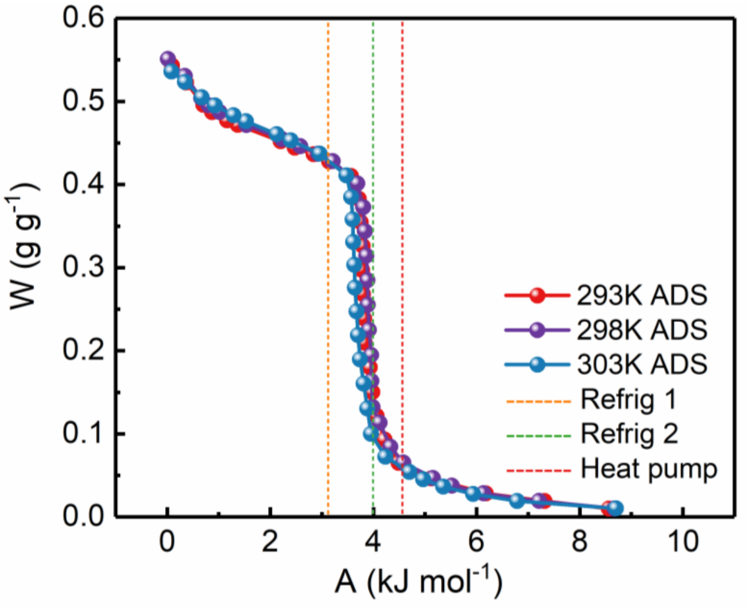

Figure S15. Characteristic curves of MIL-125-NH_2 at different temperatures determined using eq 1 and 2 The orange dotted line represents the optimal adsorption potential for refrig-1 cooling, the green dotted line for refrig-2 cooling, and the red dotted line for heat pump heating.

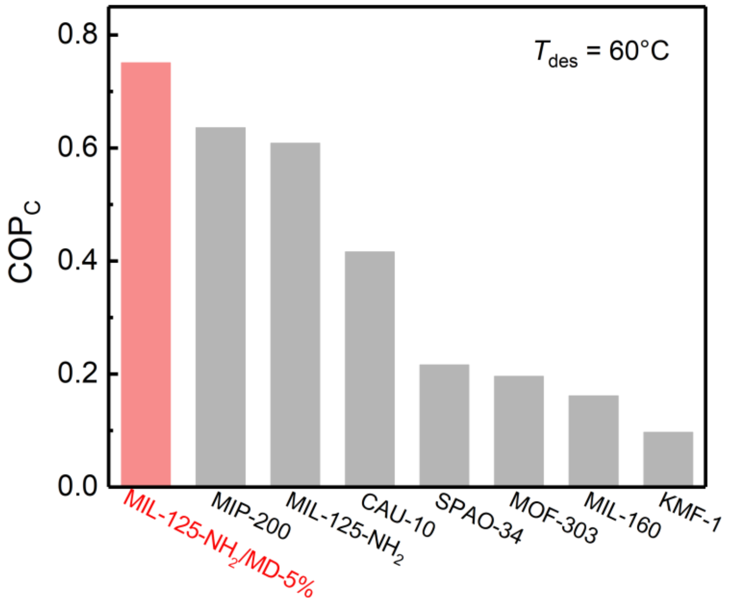

Figure S16. The comparison of COP_C of MIL-125-NH_2/MD-5% and other benchmark materials, examined under standard AC conditions (T_{ev} = 5 °C, T_{con} = 30 °C, T_{ads} = 30 °C, and T_{des} = 60 °C).

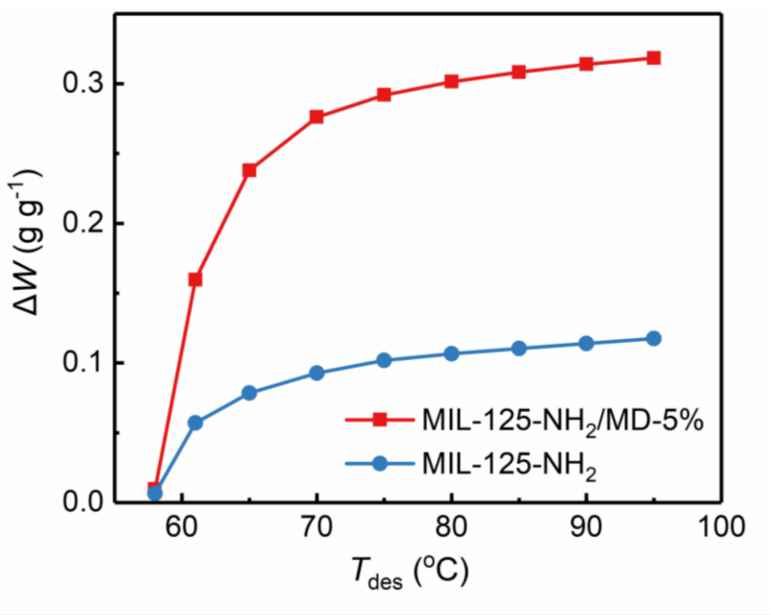

Figure S17. Working capacity (ΔW) as a function of desorption temperature (T_{des}) of MIL-125-NH_2/MD-5% and MIL-125-NH_2 for AC conditions (T_{ev} = 5 °C, T_{ads} = 30 °C, and T_{con} = 30 °C).

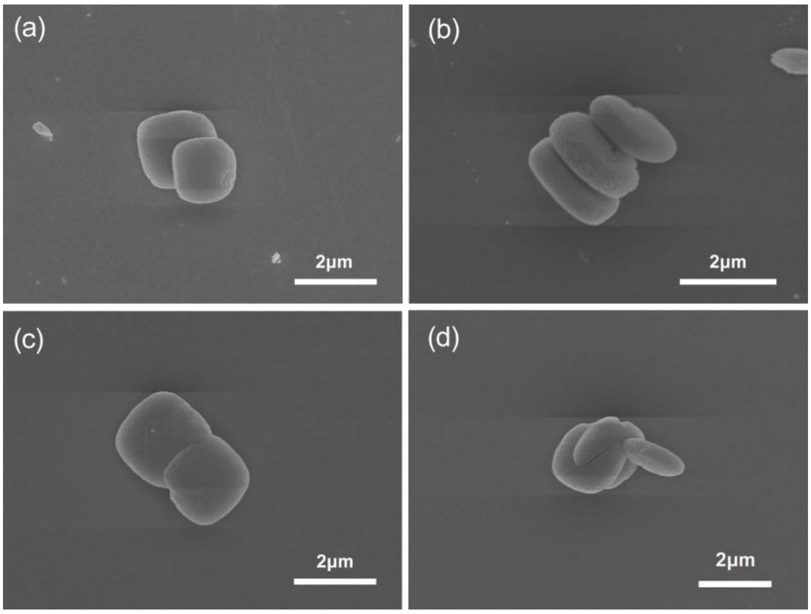

Figure S18. SEM images of (a) original MIL-125-NH_2/MD-5% and (b) after the treatments in water, (c) after soaking in pH = 1 aqueous solution, and (d) in pH = 9 aqueous solution.

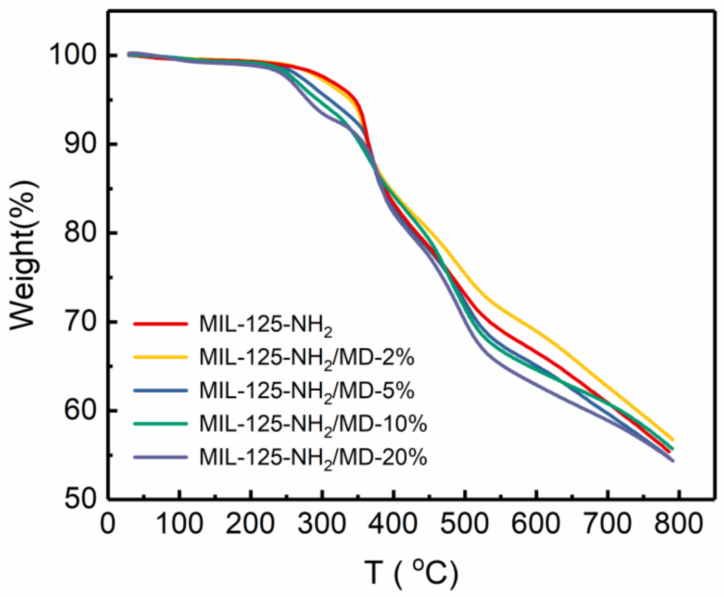

Figure S19. Thermogravimetric analysis profiles of MIL-125-NH_2, MIL-125-NH_2/MD-2%, MIL-125-NH_2/MD-5%, MIL-125-NH_2/MD-10% and MIL-125-NH_2/MD-20% (all the samples are activated).

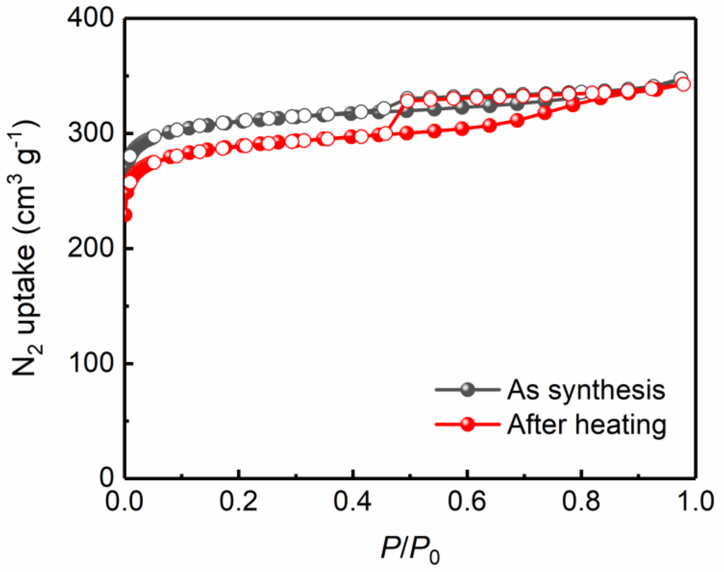

Figure S20. N_2 adsorption isotherms at 77 K of MIL-125-NH_2/MD-5% after heating to 300 °C.

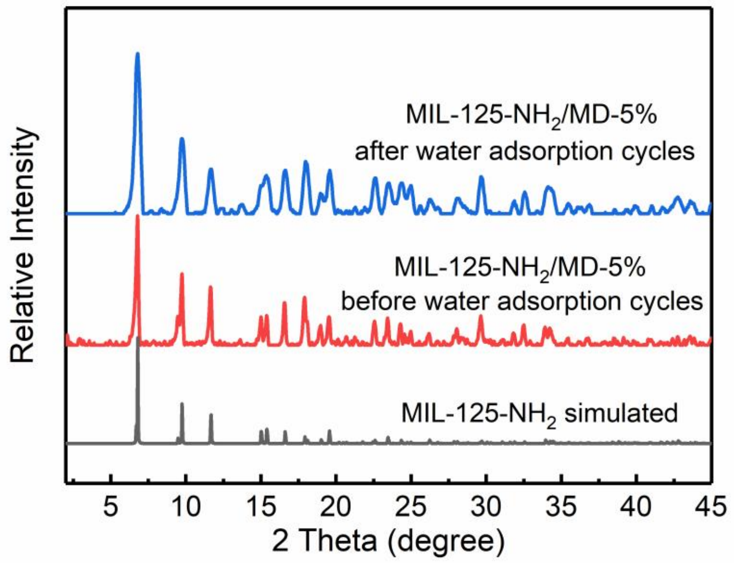

Figure S21. The PXRD patterns of MIL-125-NH_2/MD-5% before and after the water adsorption/desorption cycles compared with the simulated XRD pattern from the structure of MIL-125-NH_2.

Experimental section

Kinetic measurement and cycle test of water adsorption/desorption

The samples used to develop the kinetic measurements and cyclability tests also need to be solvent-exchanged and activated. And this two kinds of tests were developed on TA SDT 650 thermal analyzer with a humidity generator and nitrogen stream. Different humidity atmospheres are produced by adjusting the flux of dry nitrogen and moisture, and the specific humidity values are measured by a sensitive humidity sensor. Adsorption measurements were taken up in 20% RH atmosphere at 25 °C, and desorption processes were developed at 65 °C. And the water adsorption cycle tests were proceeded in 20% RH atmosphere at 25 °C in adsorption stages, and carried out at 65 °C in desorption stages.

Water adsorption enthalpy measurement

Netzsch STA 449F3 simultaneous thermal analyzer was used to measure the heat of water adsorption. First, the sample was heated to 423 K and purged with nitrogen to activate it. Then, when the temperature was maintained at 298 K, moisture was added to purge the sample and

the change of heat flux during the adsorption process was recorded.

Chemical and thermal stability tests

Four parallel samples were placed in different environments, in pure water for five days and in solutions at pH = 1 and pH = 9 for three days, respectively. After that, all the samples were filtered or picked out, and washed with dry MeOH. The integrity of crystallinity and the stability of pore structure were confirmed by PXRD and nitrogen adsorption. The thermal stability was tested by thermogravimetric analysis and various temperature PXRD patterns. Before developing the thermogravimetric analysis, samples were heated to 423 K for activation.

Working principle of adsorption refrigeration system

In general, an adsorption refrigeration cycle consists of two segments, adsorption and desorption. And each segment consists of two steps. These are briefly depicted and explained from a thermodynamic point of view by means of the cycle diagram (Figure S13).[2,10]

At first, the system is at a low temperature and pressure and the adsorbent is fully saturated. The first step is isosteric heating (I–II). Before desorption, the adsorbent chamber is isolated from the condenser and evaporator, and the pressure needs to be raised from P_{ev} to P_{con}, achieving by heating the adsorbent from T_1 to T_2. Ideally, no adsorbate is removed in this stage.

Then the second step: isobaric desorption (II–III). In this stage, the adsorbent chamber is connected to the condenser and continuously heated. After the input of higher energy (Q_{des}), the water is desorbed. This process is stopped when reaching the desorption temperature (T_{des}) and holding the minimal adsorbent loading (W_{min}). And the adsorbent completes the regeneration process. All of the desorbed water ($\Delta W = W_{max} - W_{min}$) is condensed, releasing heat at the same time (Q_{con}). In general, in order to save energy and protect the environment, industrial waste heat or solar energy is used as a source of thermal energy to complete the desorption process.

The third step: isosteric cooling (III–IV). Contrary to the first step, the pressure needs to be lowered to P_{ev} by cooling the system temperature from T_{des} to T_3, and the adsorbent chamber is again isolated from the condenser and the evaporator.

The forth step: isobaric adsorption (IV–I). In this stage, the adsorbent chamber is connected

to the evaporator, allowing water to be adsorbed. Partial water adsorbs heat from the low temperature environment through evaporation (Q_{ev}), producing useful cooling effect. The process stopped when the temperature drops to T_1, the adsorbent is saturated again (W_{max}), and the heat is released to the surrounding environment (Q_{ads}) during the adsorption process.

Calculation of the characteristic curves

At equilibrium, water adsorption capacity of porous materials such as MOF is a function of pressure and temperature. The water adsorption curves are tested at one or several fixed temperatures to evaluate and compare the water adsorption properties of materials. In order to character and describe conveniently, Polanyi adsorption potential is introduced to reduce the number of variables, which is the inverse of the molar Gibbs free energy of adsorption, defined as:

$$A = -\Delta G = RT \ln\left(\frac{P_0(T)}{P}\right) \quad (1)$$

P_0 is the temperature-dependent vapor pressure of water. R is the universal gas constant.

At the same time, the adsorption amount should be expressed in terms of the volume of adsorbed water molecules, defined as:

$$W = \frac{q(P,T)}{\rho_{liq}^{wf}(T)} \quad (2)$$

Here, q is the mass of the adsorbate.

In general, the adsorption data measured at different temperatures can be reduced to a characteristic curve. This means that each combination of pressure and temprature (P, T) can be converted to a single adsorption potential A, and the adsorption volume W can be easily determined by interpolating the characteristic curve. The adsorption curves of MIL-125-NH_2/MD-5% were tested at 293 K, 298 K and 303 K respectively, and they were converted into characteristic curve by simple calculation, and all the characteristic curves were almost exactly coincident (Figure S14). The same is true for MIL-125-NH_2 (Figure S15).

Calculation the isosteric enthalpy of adsorption

Water adsorption is an exothermic process, and the isosteric enthalpy of adsorption can be calculated using a form of the Clausius–Clapeyron equation given by[11,12]

$$\Delta_{\text{ads}}H_W = R\left(\frac{\partial \ln P}{\partial(1/T)}\right)_W \tag{3}$$

Here, $\Delta_{\text{ads}}H_W$ represents the isosteric enthalpy of adsorption (kJ mol^{-1}), P represents pressure (bar), T represents temperature (K).

And also given by:

$$\Delta_{\text{ads}}H_W = -\frac{RT_1T_2}{T_2-T_1}\ln\frac{P_2}{P_1} \tag{4}$$

T_1 and T_2 are two temperatures in isotherm, and P_1 and P_2 are pressures at T_1 and T_2 , respectively, for a given uptake.

Calculation of the coefficient of performance

The coefficient of performance for cooling (COP_C), which is the commonly adopted parameter to evaluate the cooling effect, defined as the useful energy output divided by the energy required for input. It is writen as:

$$COP_C = \frac{Q_{\text{ev}}}{Q_{\text{regen}}} \tag{5}$$

Here, Q_{ev} is the energy taken up in the evaporator, and Q_{regen} is the energy of adsorbents regeneration.

According to working principle, the energy required in stage I-II and stage II-III is the desorption energy (Q_{des}), that is, the energy required for the regeneration of the adsorbent, while the energy released in stage III-IV and satge IV-I is the adsorption energy (Q_{ads}).

The energy required for each stage is expressed in the following equations:

Isosteric heating (I-II):

$$Q_{\text{I-II}} = \int_{T_{\text{ads}}}^{T_2} c_p^{eff}(T)dT + \int_{T_{\text{ads}}}^{T_2} \rho_{liq}^{wf} W_{\max} c_p^{wf}(T)dT \tag{6}$$

Isobaric desorption (II−III):

$$Q_{\text{II-III}} = \int_{T_2}^{T_{\text{des}}} c_p^{eff}(T)dT + \int_{T_2}^{T_{\text{des}}} \rho_{liq}^{wf} \frac{W_{\max}+W_{\min}}{2} c_p^{wf}(T)dT - Q_{\text{sorption}} \tag{7}$$

Isosteric cooling (III−IV):

$$Q_{\text{III-IV}} = \int_{T_{\text{des}}}^{T_4} c_p^{eff}(T)dT + \int_{T_{\text{des}}}^{T_4} \rho_{liq}^{wf} W_{\max} c_p^{wf}(T)dT \quad (8)$$

Isobaric adsorption (IV−I):

$$Q_{\text{IV-I}} = \int_{T_4}^{T_{\text{ads}}} c_p^{eff}(T)dT + \int_{T_4}^{T_{\text{ads}}} \rho_{liq}^{wf} \frac{W_{\max} + W_{\min}}{2} c_p^{wf}(T)dT + Q_{\text{sorption}} \quad (9)$$

Thus,

$$Q_{\text{regen}} = Q_{\text{I-II}} + Q_{\text{II-III}} \quad (10)$$

$$Q_{\text{ads}} = Q_{\text{I-II}} + Q_{\text{II-III}} \quad (11)$$

The energy required for water evaporation in the evaporator (Q_{ev}) is calculated by:

$$Q_{\text{ev}} = -\frac{\Delta_{\text{vap}} H(T_{\text{ev}}) \rho_{liq}^{wf} m_{\text{sorbent}} \Delta W}{M_{\text{W}}} \quad (12)$$

The energy released by the condenser (Q_{con}) is calculated by:

$$Q_{\text{con}} = \frac{\Delta_{\text{vap}} H(T_{\text{con}}) \rho_{liq}^{wf} m_{\text{sorbent}} \Delta W}{M_{\text{W}}} \quad (13)$$

The energy released during adsorption of the working fluid (Q_{sorption}) can be written as:

$$Q_{\text{sorption}} = \frac{1}{M_{\text{W}}} \int_{W_{\min}}^{W_{\max}} \rho_{liq}^{wf} \Delta_{\text{ads}} H(W) \mathrm{d}W \quad (14)$$

List of symbols

Latin letters

Q	energy, kJ mol^{-1}
T	temperature, K
P	pressure, kPa
W	volumetric adsorption capacity, mL(liq) g^{-1}
q	gravimetric adsorption capacity, g g^{-1}
ΔW	working capacity, mL(liq) g^{-1}
A	adsorption potential, kJ mol^{-1}
G	Gibbs free energy, kJ mol^{-1}
R	ideal gas constant, J K^{-1} mol^{-1}
P/P_0	relative pressure
P_0	saturation pressure

c_p heat capacity, J g^{-1} K^{-1}/ (J mol^{-1} K^{-1})

m mass, g

M_w molar mass, g mol^{-1}

Greek symbols

$\Delta_{ads}H$ enthalpy of adsorption, kJ mol^{-1}

$\Delta_{vap}H$ enthalpy of evaporation, kJ mol^{-1}

ρ liquid density of the adsorbate, g cm^{-3}

Subscripts

ev evaporation

con condensation

ads adsorption

des desorption

regen regeneration

liq liquid

min minimum

max maximum

Superscripts

eff effective

wf working fluid

Abbreviation

refrig refrigeration

References

[1] M. F. de Lange, K. J. F. M. Verouden, T. J. H. Vlugt, J. Gascon, F. Kapteijn, *Chem. Rev.* **2015**, *115*, 12205.

[2] F. Jeremias, V. Lozan, S. K. Henninger, C. Janiak, *Dalton Trans.* **2013**, *42*, 15967.

[3] S. Wang, J. S. Lee, M. Wahiduzzaman, J. Park, M. Muschi, C. Martineau-Corcos, A. Tissot, K. H. Cho, J. Marrot, W. Shepard, G. Maurin, J. Chang, C. Serre, *Nat. Energy* **2018**, *3*, 985.

[4] K. H. Cho, D. D. Borges, U. H. Lee, J. S. Lee, J. W. Yoon, S. J. Cho, J. Park, W. Lombardo, D. Moon, A. Sapienza, G. Maurin, J. Chang, *Nat. Commun.* **2020**, *11*, 5112.

[5] A. Cadiau, J. S. Lee, D. Damasceno Borges, P. Fabry, T. Devic, M. T. Wharmby, C. Martineau, D. Foucher, F. Taulelle, C. Jun, Y. K. Hwang, N. Stock, M. F. De Lange, F. Kapteijn, J. Gascon, G. Maurin, J. Chang, C. Serre, *Adv. Mater*. **2015**, *27*, 4775.

[6] D. Lenzen, P. Bendix, H. Reinsch, D. Fröhlich, H. Kummer, M. Möllers, P. P. C. Hügenell, R. Gläser, S. Henninger N. Stock, *Adv. Mater.* **2018**, *30*, 1705869.

[7] D. Fröhlich, E. Pantatosaki, P. D. Kolokathis, K. Markey, H. Reinsch, M. Baumgartner, M. A. V. D. Veen, D. E. D. Vos, N. Stock, G. K. Papadopoulos, S. K. Henninger, C. Janiak, *J. Mater. Chem. A* **2016**, *4*,11859.

[8] N. Hanikel, M. S. Prévot, F. Fathieh, E. A. Kapustin, H. Lyu, H. Wang, N. J. Diercks, T. G. Glover, O. M. Yaghi, *Acs Cent. Sci.* **2019**, *5*, 1699.

[9] B. N. Truong, D. D. Borges, J. Park, J. S. Lee, D. Jo, J. Chang, S. J. Cho, G. Maurin, K. H. Cho, U. Lee, *Adv. Sci.* **2023**, 2301311.

[10] X. Liu, X. Wang, F. Kapteijn, *Chem. Rev.* **2020**, *120*, 8303.

[11] M. Pons, F. Poyelle, *Int. J. Refrig.* **1999**, *22*, 27.

[12] M. Pons, F. Meunier, G. Cacciola, R. E. Critoph, M. Groll, L. Puigjaner, B. Spinner, and F. Ziegler, *Int. J. Refrig.* **1999**, *22*, 5.
